# Reduced Angiopoietin-Like 4 Expression in Multiple Sclerosis Lesions Facilitates Lipid Uptake by Phagocytes via Modulation of Lipoprotein-Lipase Activity

**DOI:** 10.3389/fimmu.2019.00950

**Published:** 2019-05-03

**Authors:** Alwin Kamermans, Merel Rijnsburger, Ananya Chakraborty, Susanne van der Pol, Helga E. de Vries, Jack van Horssen

**Affiliations:** Department of Molecular Cell Biology and Immunology, MS Center Amsterdam, Amsterdam Neuroscience, Amsterdam UMC, Vrije Universiteit Amsterdam, Amsterdam, Netherlands

**Keywords:** angiopoietin-like 4, multiple sclerosis, phagocytes, astrocyte, lipoprotein-lipase, remyelination

## Abstract

Multiple sclerosis (MS) is a chronic inflammatory disorder of the central nervous system (CNS) characterized by the presence of focal demyelinated plaques. Sufficient clearance of myelin and cellular debris is one of the requirements for proper tissue repair and remyelination. The mechanisms underlying the clearance of such debris by phagocytes are not fully understood, but recent findings suggest a prominent role for lipoprotein-lipase (LPL) in this process. Here, we demonstrate that angiopoietin-like 4 (ANGPTL4), a potent inhibitor of LPL, is abundantly expressed in astrocytes in control white matter tissue and its expression is markedly reduced in active MS lesions. We provide evidence that ANGPTL4 inhibits the uptake of myelin-derived lipids by LPL-immunoreactive phagocytes. Taken together, our data suggest that the strong reduction in astrocytic ANGPTL4 expression in active demyelinating MS lesions enables phagocytes to adequately clear myelin debris, setting the stage for remyelination.

## Introduction

MS is an inflammatory demyelinating disease characterized by massive infiltration of monocyte-derived macrophages into the central nervous system (CNS). Infiltrated macrophages, as well as brain-resident activated microglia, produce a variety of cytotoxic factors and cytokines and thereby contribute to CNS damage and associated neurodegeneration. Macrophage depletion in an experimental MS animal model significantly reduces clinical symptoms underscoring the pathogenic role of these cells (1). However, macrophages and microglia also exert neuroprotective properties. Notably, intravenous administration of anti-inflammatory macrophages or microglia reduced clinical signs in the MS animal model experimental autoimmune encephalomyelitis (EAE) (2, 3). A well-known mechanism by which phagocytes promote regeneration is via clearance of myelin debris. Removal of damaged myelin components and apoptotic cells is a requisite for remyelination (4, 5). Although the cellular mechanisms involved in clearance of myelin debris by phagocytes are poorly understood, evidence is emerging that lipoprotein-lipase (LPL), an enzyme involved in lipid-processing, plays an important role during initiation of remyelination (4, 6). Activity of this enzyme is significantly increased in brain tissue of the EAE model at the point when clinical symptoms start to decrease and shows to be involved in lipid and lipoprotein uptake in microglia (6). This suggests that LPL-expressing phagocytes might contribute to repair and support remyelination through the clearance and reuptake of lipid debris. Regulation of phagocyte function and activity is under control of astrocytes (7) and recently, our group described the presence of, angiopoietin-like 4 (ANGPTL4), a potent inhibitor of LPL, in astrocytes (8). We here provide evidence for decreased astrocytic expression of ANGPTL4 within active inflammatory MS lesions. We show that the cellular communication between astrocytes and macrophages is necessary for the downregulation of astrocytic ANGPTL4 expression. Furthermore, we show that ANGPTL4-mediated inhibition of LPL activity reduced myelin-lipid uptake by phagocytes, without affecting phagocytosis. Taken together, our findings suggest that the strong reduction in astrocytic ANGPTL4 expression in active demyelinating MS lesions enables LPL-immunopositive phagocytes to adequately clear myelin debris, paving the way for remyelination.

## Materials and Methods

### Immunohistochemistry

Blocks of formalin-fixed paraffin-embedded post-mortem brains samples were obtained from the VUmc MS Centrum Amsterdam and the Netherlands Brain Bank from 12 MS patients and 3 non-neurological controls. Detailed clinical data are summarized in Table 1. Sections (5 μm) from each block were cut with a microtome. Sections were deparaffinized in xylene and rehydrated through graded alcohol into distilled water. Antigen retrieval was performed using citrate buffer pH 6.0 (0.01M) at 100°C for 10 min. Sections were incubated overnight with appropriate antibodies (see Table 2) in phosphate buffered saline (PBS) supplemented with 1% bovine serum albumin (BSA) and subsequently stained with the EnVision horseradish peroxidase kit (Dako, K4061, Belgium) for 30 min at room temperature and followed by 3,3′diaminobenzidine-tetrachloridedihydrate (DAB). Between incubation steps, the sections were thoroughly washed with PBS. After a short rinse in tap water the sections were incubated with haematoxylin for 1 min and intensely washed with tap water for 10 min. Finally, the sections were dehydrated with ethanol followed by xylene and mounted with Entellan (Merck, #107960, Germany).

**Table 1:** Clinical data of MS patients and non-neurological controls.

**Case**	**Age (years)**	**Type of MS**	**Gender**	**Post-mortem delay (h:min)**	**Disease duration (years)**	**Cause of death**
Ctrl 1	87	NA	F	07:20	NA	Cachexia and dehydration
Ctrl 2	83	NA	M	05:15	NA	Unknown
Ctrl 3	51	NA	F	05:36	NA	Unknown
MS 1	61	RR	F	10:55	Unknown	Sepsis
MS 2	39	RR/SP	F	08:30	8	Ileus
MS 3	75	SP	M	22:20	35	Urosepsis
MS 4	48	SP	F	09:20	24	Pneumonia
MS 5	56	RR	F	08:55	20	Legal euthanasia
MS 6	61	RR	F	10:55	Unknown	Sepsis
MS 7	41	PP	M	07:23	14	Urosepsis and pneumonia
MS 8	49	RR	M	08:00	25	Pneumonia
MS 9	51	SP	M	11:00	18	Infection
MS 10	44	SP	M	10:15	21	Unknown
MS 11	57	SP	F	08:40	27	Respiratory insufficiency
MS 12	54	PP	M	08:15	12	Legal euthanasia

*MS, multiple sclerosis; SP, secondary progressive MS; RR, relapse remitting MS; PP, primary progressive MS; ND, non-determined; NA, non-applicable; M, male; F, female*.

**Table 2:** Antibody details.

**Antigen**	**Species**	**Dilution**	**Manufacturer**	**Cat. number**
HLA-DR	Mouse	1:1000	eBioscience	14-9956-82
PLP	Mouse	1:3000	Rio-rad	MCA839G
ANGPTL4	Rabbit	1:300	Abcam	ab115798
GFAP-cy3	Mouse	1:300	Sigma	C9205
GFAP	Rabbit	1:250	Chemicon	ab1980
LPL	Mouse	1:100	Abcam	ab21356
Iba1	Goat	1:100	Abcam	Ab5076

*HLA-DR, human leukocyte antigen-DR; LPL, Lipoprotein-lipase; ANGPTL4, Angiopoietin-like 4; GFAP, glial fibrillary acidic protein; PLP, proteolipid protein; Iba1, ionized calcium binding adaptor molecule 1*.

For cellular localization sections were incubated overnight with antibodies applied simultaneously at 4°C. After washing with PBS, secondary antibodies consisting of donkey-anti-mouse Alexa Fluor 488 (1:200, Abcam, ab150105, UK), rabbit-anti-goat Alexa Fluor 647 (1:200, Abcam, ab150143, UK), or goat-anti-rabbit Alexa Fluor 647 (1:200, Abcam, ab150079, UK) were applied for 1 h at room temperature. Fluorescent preparations were embedded and analysis was performed with a Leica TCS SP8 confocal laser-scanning microscope (Leica Microsystems, Heidelberg, Germany). GFAP and ANGPTL4 fluorescence intensity were measured by a blinded observer using ImageJ software (freely available from: U.S. National Institutes of Health, Bethesda, MD, USA). Per slide, 3 independent locations were analyzed. Relative ANGPTL4 intensity to GFAP was calculated by dividing ANGPTL4 intensity over GFAP intensity. Next, intensity was normalized to the relative intensity of ANGPTL4 over GFAP in NAWM slides.

### Human Astrocyte Culture

Human astrocytoma cells (U373) were cultured in Dulbecco's modified Eagle's medium (DMEM)/F12 (Life Technologies, #11320033, USA) containing 10% fetal bovine serum (FBS; Biowest, S1810-500, USA), and penicillin/streptomycin (50 mg/ml; Life Technologies, #15140122, USA) in 5% CO_2_ at 37°C.

### RNA Isolation and Real-Time Quantitative PCR

RNA was isolated using Trizol (Invitrogen, Carlsbad, CA, USA) according to manufacturer's protocol. mRNA concentration and quality (OD _280/260_ ratios of 1.8 or higher) was measured using Nanodrop (Nanodrop Technologies, USA). cDNA syntheses was performed using the Reverse Transcription System kit (Applied Biosystems, #4368814, USA) following manufacture's guidelines. Expression was assessed by quantitative RT-PCR using SYBR Green Power mix (Applied Biosystems, #4367659, USA). All primer sequences are listed in Table 3. qPCR reaction was performed using the Step-one (Applied Bioscience, USA) Real-Time PCR System with the following program: 2 min at 50°C, 10 min at 95°C and then 40 cycles of 15 s at 95 °C and 1 min at 60°C. mRNA expression levels were quantified using the 2^−ΔΔ*CT*^ method as described in (9).

**Table 3:** Primer sequences.

**Target gene**	**Species**	**Forward primer**	**Reverse primer**
P2X7	Human	GAA CAA TAT CGA CTT CCC CGG	TTA TCG CCT GTT TCT CGG AAG
CD40	Human	CAA ATA CTG CGA CCC CAA CCT A	TTT CTG AGG TGC CCT TCT GCT
CD206	Human	GTC TTG GGC CAC AGG TGA A	AAG GCG TTT GGA TAG CCA CA
P2Y12	Human	ACC AGA GAC TAC AAA ATC ACC C	AGA AAA TCC TCA TCG CCA GG
LPL	Human	CGA GCG CTC CAT TCA TCT CT	CCA GAT TGT TGC AGC GGT TC
ANGPTL4	Human	ATG GCT CAG TGG ACT TCA AC	GCT ATG CAC CTT CTC CAG AC
18S	Human	TAC CAC ATC CAA GGA AGG CAG CA	TGG AAT TAC CGC GGC TG CTG GCA
HMBS	Human	CAC GAT CCC GAG ACT CTG CT	TAC TG GCA CAC TGC AGC CTC

### Myelin Isolation

Myelin was isolated and labeled as described previously (10). In short, human CNS white matter tissue was obtained from three healthy controls. Myelin was isolated by homogenization and sucrose gradient centrifugation after which protein concentration was assessed by the bicinchoninic acid method. Isolated myelin was subsequently labeled with NHS-activated Atto633.

### Human Macrophages Cultures

Human blood monocytes were isolated from buffy coats of healthy donors (Sanquin Blood Bank, The Netherlands) using Ficoll (Lymphoprep™, Axis-Shield, #1114544 Norway) and subsequent Percoll density gradient centrifugation. Monocytes were differentiated into macrophages in culture medium (IMDM, Gibco) containing 10% FBS, penicillin (100IU/ml), streptomycin (50 mg/ml) and 50 ng/ml MCSF, for 6 days at 37°C, 5% CO_2_. The alternatively activated macrophages phenotype was induced by culturing M-CSF differentiated macrophages in the presence of 10 ng/ml human IL-4 (ImmunoTools, #13340043, Germany) for 48 h as described before by (11).

### Macrophage/Astrocyte Coculture

Human monocytes were cultured on top of a transwell filter (Corning, #3421, USA) with a pore size of 0.3 μm and differentiated into macrophages as described above. Human astrocytoma cells (U373) were plated in a culture plate. After cell adhesion, the transwell filter containing macrophages was transferred to the culture plate containing astrocytes. After 24 h at 37°C in 5% CO_2_, the transwell filter containing macrophages was discarded and mRNA from astrocytes was isolated as described above.

### Oil-Red-O Staining

Alternatively activated macrophages were cultured on glass coverslips and fixed with 4%PFA for 10 min. Oil-Red-O stock solution was prepared by dissolving in 0.25 g Oil-Red-O (Sigma-Aldrich, #O0265, USA) in 50 ml isopropanol. Oil-Red-O working solution was prepared by mixing 3 parts Oil-Red-OO stock solution with 2 parts dH2O, followed by filtration. Coverslips were immersed in Oil-Red-O working solution for 10 min, followed by two rinses with 60% isopropanol and one rinse with dH2O. Nuclear staining was performed using Haematoxylin for 5 min followed by several rinses with dH2O. Quantification of Oil-Red-O staining was performed by extracting Oil-Red-O using 100% isopropanol for 5 min. Absorbance was subsequently measuring at 492 nm. 100% isopropanol was used as background control.

### Myelin Phagocytosis Assay

For phagocytosis experiments, alternatively activated macrophages were exposed to 5 μg/ml atto633-labeled myelin for 4 h at 37°C in 5% CO_2_, with or without addition of 1 μg/ml ANGPTL4 (R&D systems, 4487-AN, USA) or 10 μM Cytochalasin D (Sigma-Aldrich, C8273, USA). Subsequently, cells were thoroughly washed with PBS to remove extracellular myelin. Phagocytosis of fluorescent myelin particles was quantified using intracellular FACS analysis (Calibur flow cytometer, Becton & Dickinson, USA).

### Lipoprotein Lipase Activity (LPL) Assay

Lipoprotein lipase activity on alternatively activated monocyte derived macrophages was measured using a fluorometric assay kit (Abcam, ab204721, UK) according to the manufacturer's instructions.

### Statistics

All data reflect mean ± SEM and all comparisons were statistically tested in GraphPad Prism 5.0. For comparing two experimental groups with normal distribution, unpaired two-tailed Student's *t*-tests was used. For comparing two experimental groups without normal distribution Mann–Whitney *U*-test was used. One-way analysis of variance (ANOVA) was used to compare more than two groups.

## Results

### Decreased Astrocytic ANGPTL4 Expression in Multiple Sclerosis Lesions

**Figure 1:**
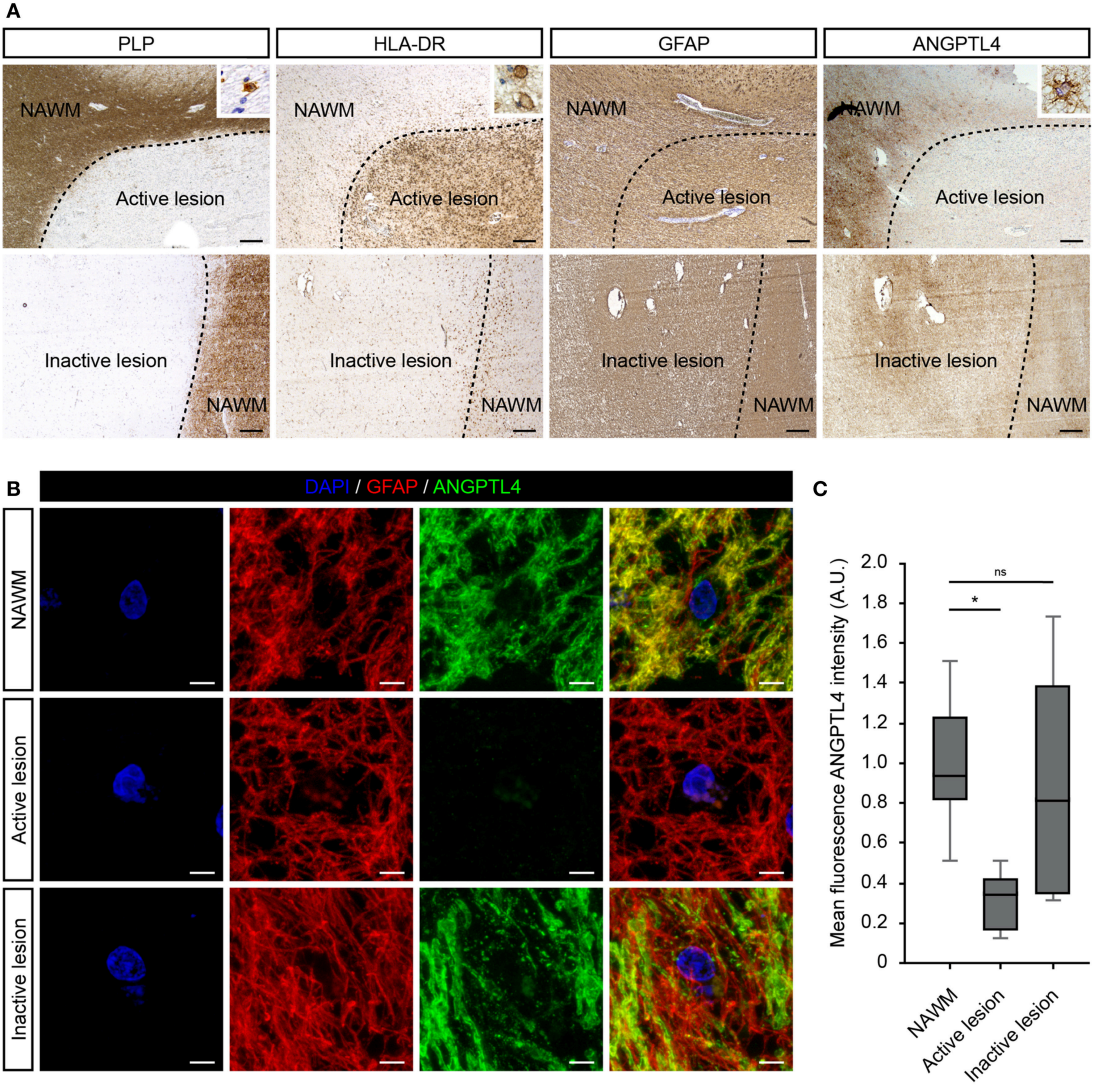
ANGPTL4 expression is strikingly reduced in active MS lesions. **(A)** Active white matter lesions are characterized by loss of PLP and the presence of HLA-DR positive cells containing PLP-positive fragments (insert). Inactive white matter lesions display a loss of PLP immunoreactivity and absence of HLA-DR positive cells. Active white matter lesions display a marked decreased expression of ANGPTL4 compared to normal appearing white matter and inactive lesions. ANGPTL4 is predominantly localized to astrocytes (insert) (scale bar = 500 μm). **(B)** Double immunofluorescence labeling shows co-localization of ANGPTL4 (green) with GFAP-immunoreactive astrocytes (red) (scale bar = 5 μm). **(C)** Quantification of fluorescence intensity of ANGPTL4 in NAWM, active lesions and inactive lesions shows downregulation of ANGPTL4 in active MS lesions (One-way ANOVA, *N* = 6 for NAWM, 5 for active lesion and 4 for inactive lesion). *p < 0.05.

First, we investigated the cellular expression of ANGPTL4 in well-characterized MS lesions. Active white matter lesions were identified by the absence of myelin (proteolipid protein) and the presence of MHC class II^+^ cells with some MHC class II cells containing myelin proteins. Inactive lesions are characterized with a demyelinated core with little evidence of ongoing inflammation. We observed no clear differences in cellular expression nor intensity of ANGPTL4 immunoreactivity comparing control white matter with normal appearing white matter (NAWM). ANGPTL4 was found to be expressed in normal appearing white matter by cells with an astrocytic morphology. Interestingly we observed a marked decreased expression of ANGPTL4 in active MS lesions compared to NAWM, while in inactive lesions the expression of ANGPTL4 displayed similar immunoreactivity as observed in NAWM (Figure 1A). To confirm the cellular source of ANGPTL4, we performed co-localization studies which confirmed that ANGPTL4 is predominantly expressed by glial fibrillary acidic protein (GFAP)-positive astrocytes (Figure 1B). Fluorescence intensity of ANGPTL4 was measured in NAWM, active and inactive lesions. These quantitative analyses showed that ANGPTL4 immunoreactivity is consistently reduced in active MS lesions compared to surrounding NAWM and to inactive lesions (Figure 1C).

### Lipoprotein-Lipase Is Expressed by Iba-1 Positive Cells in MS Lesions

**Figure 2:**
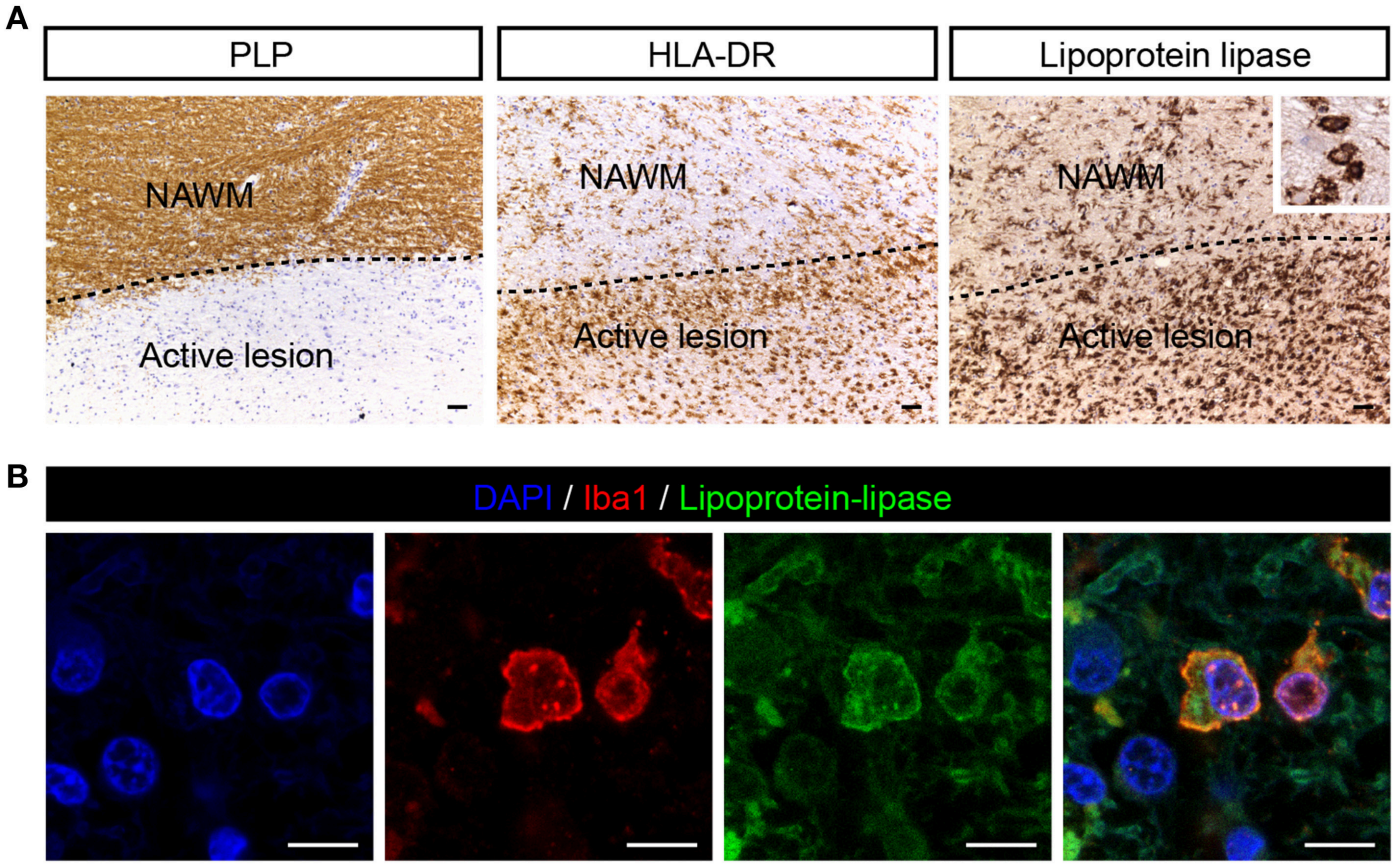
LPL is expressed on iba1 positive cells in active MS lesion. **(A)** Active white matter lesion is characterized by loss of PLP. Active lesions showed enhanced LPL immunoreactivity in microglia/macrophages (insert) (scale bar = 50 μm). **(B)** Double immunofluorescent labeling shows presence of lipoprotein-lipase (green) positive Iba1 (red) cells in MS lesions (scale bar = 10 μm).

Since ANGPTL4 is a known inhibitor of lipoprotein-lipase (LPL), we next analyzed the cellular distribution of LPL in MS brain specimens. LPL was weakly expressed in NAWM and abundantly expressed in active lesions, localized to cells with the morphological appearance of macrophages (Figure 2A). Immunofluorescent double staining with Allograft inflammatory factor 1 (iba1, macrophage/microglia marker) confirmed the cellular localization of LPL in macrophages/microglia (Figure 2B). Taken together, ANGPTL4 expression is virtually absent in astrocytes in active lesions, while LPL, the target of ANGPTL4, is expressed by Iba1 positive phagocytes in active lesions.

### Crosstalk Between Phagocytes and Astrocyte Underlies Downregulation of ANGPTL4

**Figure 3:**
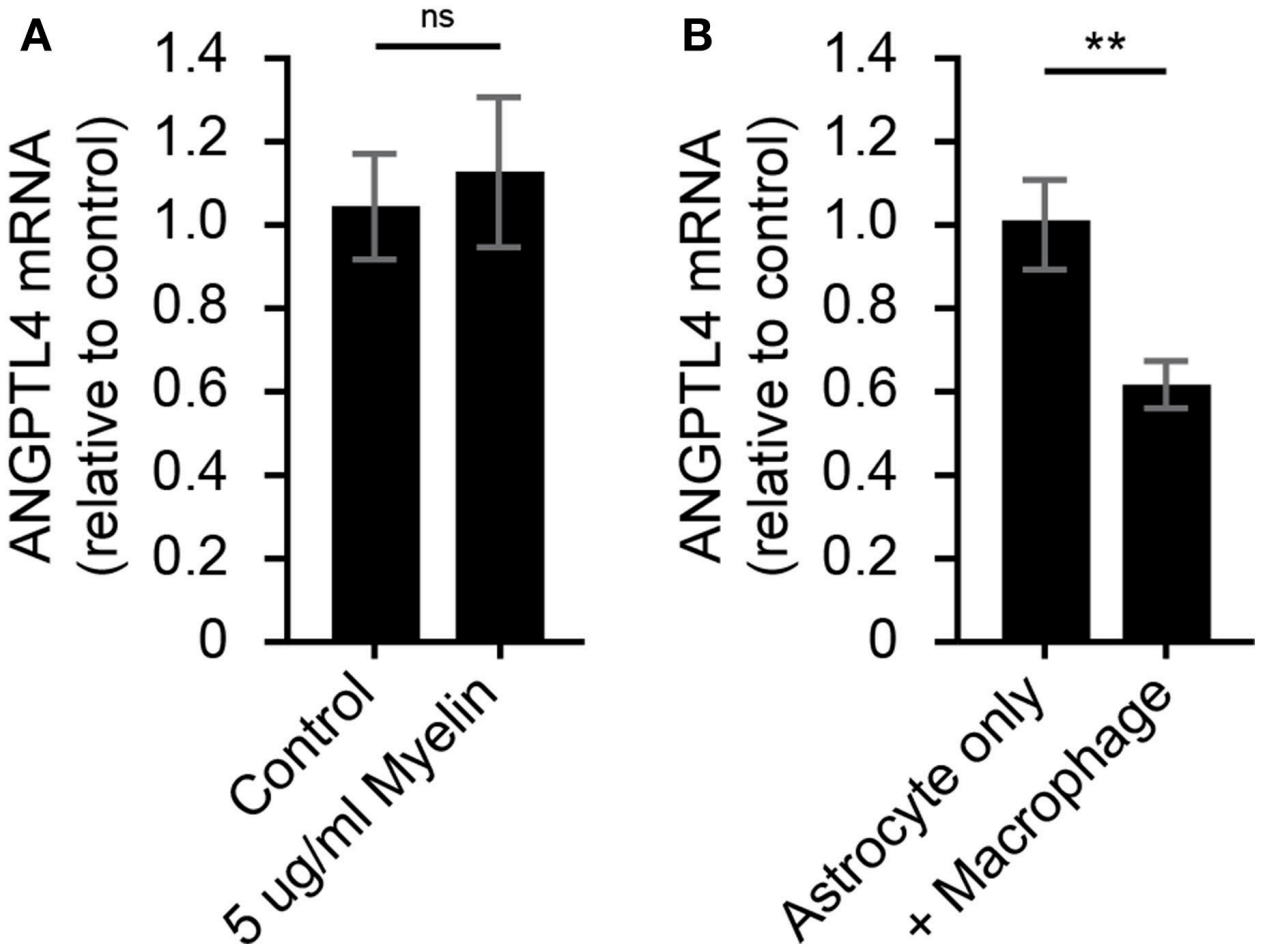
Astrocytic ANGPTL4 expression is not influenced by myelin, but is by crosstalk with macrophages. **(A)** Astrocytes express ANGTPL4 at similar levels under normal conditions compared to 24 h treatment with myelin (Mann Whitney, N=8 for control and 11 for myelin treatment). Expression of ANGPTL4 is significantly reduced in astrocytes cultures in the presence of alternatively activated macrophages (Student's *t*-test, *N* = 7) **(B)**. **p < 0.01.

To determine what underlies the observed decrease in astrocytic ANGPTL4 expression, human astrocytes were exposed to myelin for 24 h. Exposure to myelin did not affect ANGPTL4 expression in astrocytes (Figure 3A). Based on the co-occurrence of LPL-positive phagocytes and ANGPTL4-deficient astrocytes in active lesions, we hypothesized that macrophages might be responsible for the observed loss of astrocytic ANGPTL4. Astrocytes cultured in the presence of activated macrophages showed a significant decreased expression of ANGPTL4 compared to astrocytes cultured in the absence of macrophages (Figure 3).

### ANGPTL4 Inhibits (Myelin) Lipid Uptake via Modulation of LPL Activity

**Figure 4:**
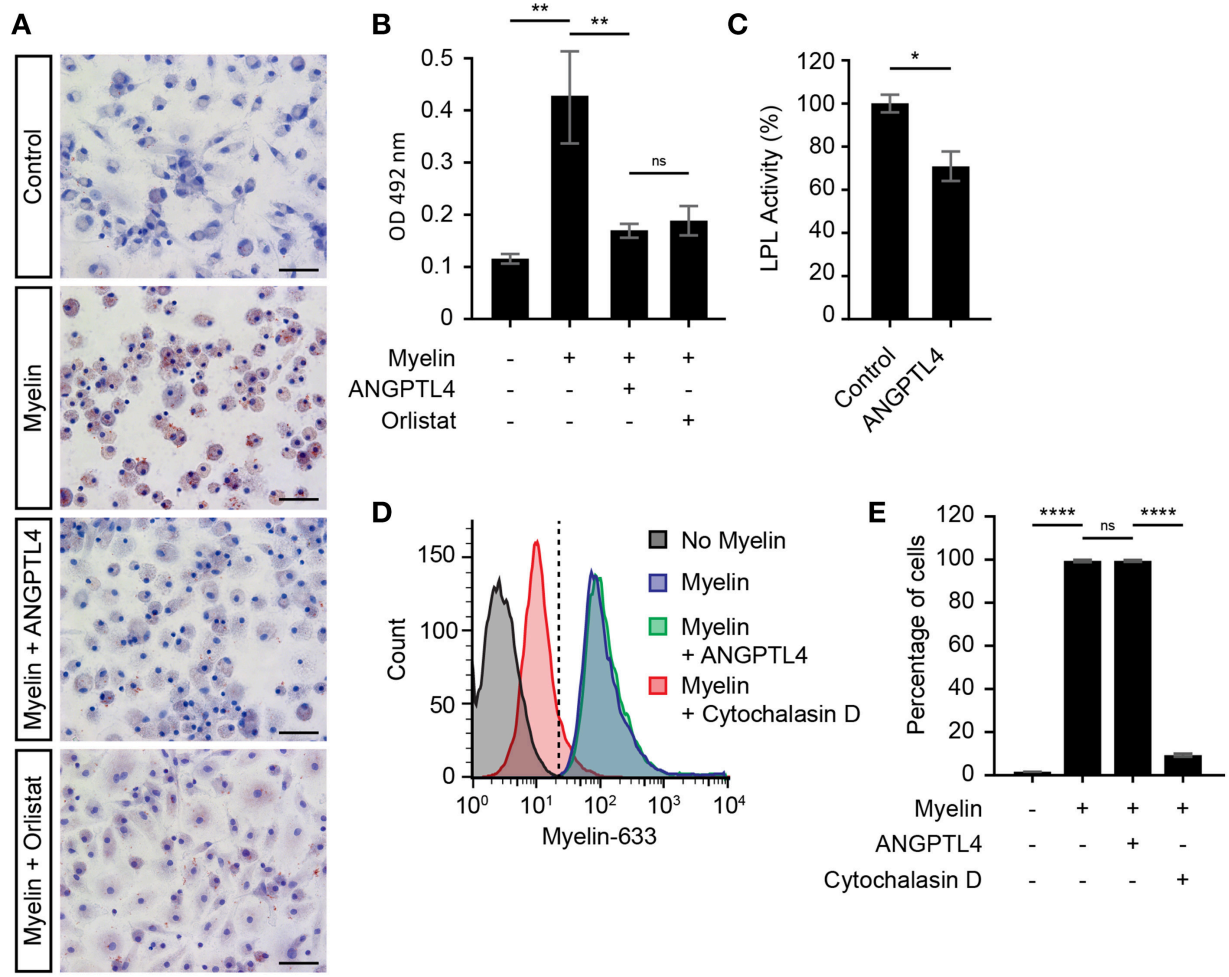
ANGPTL4 inhibits uptake of myelin derived lipids but not myelin phagocytosis. **(A)** Increased Oil-Red-O staining of monocyte derived macrophages incubated for 4 hr with myelin, compared to control and to treatment of myelin together with recombinant Angptl4 or Orlistat. (scale bar = 50 μm) **(B)** Quantification of Oil-Red-O staining. (One-way ANOVA, *N* = 5) **(C)** ANGPTL4 inhibits macrophage LPL activity as determined by LPL activity assay. (Mann Whitney, *N* = 4) **(D,E)** ANGPTL4 treatment did not influence phagocytosis of myelin as determined by flow cytometry (One-way ANOVA, *N* = 5). *p < 0.05,**p < 0.01, ****p ≤ 0.0001.

Recent reports have highlighted the pivotal role of microglial LPL in remyelination (4, 6, 12), and it is thought that microglial LPL is able to process myelin after which the myelin derived lipids can be taken up via scavenger receptors expressed on microglia (13). Here we investigated whether LPL expression on macrophages is indeed involved in myelin uptake and tested the hypothesis that ANGPTL4 inhibits this uptake by decreasing LPL activity. Macrophages were exposed to myelin, in the absence or presence of ANGPTL4. We first analyzed whether myelin treatment induced lipid accumulation in macrophages. As shown by Oil-Red-O staining, we observed increased lipid accumulation after myelin treatment (Figures 4A,B). Macrophages that were treated with ANGPTL4 peptide during exposure to myelin displayed decreased Oil-Red-O staining, demonstrating that ANGPTL4 inhibits (myelin) lipid uptake. To see if a well-known LPL inhibitor also causes a reduction in Oil-Red-O staining, we treated the cells with the LPL inhibitor Orlistat. Treatment with Orlistat resulted in similar reduction as ANGPTL4. Using an LPL activity assay, we confirmed that ANGPTL4 causes a significant decrease in LPL activity in macrophages compared to non ANGPTL4 treated macrophages (Figure 4C). To confirm that ANGPTL4 inhibits myelin uptake by blocking LPL activity and not by affecting phagocytosis, we exposed macrophages to atto633-labeled myelin, and quantified the amount of atto633-labeled myelin inside the cells by FACS (Supplementary Figure 1). Phagocytosis of atto633-labeled myelin was not inhibited by ANGPTL4, while treatment with Cytochalasin D, which blocks phagocytosis, completely prevented the myelin phagocytosis by macrophages (Figures 4D,E). Our findings suggest that macrophages utilize LPL in the process of myelin clearance. In addition, these findings show that astrocyte derived ANGPTL4 inhibits LPL activity, leading to decreased myelin derived lipid uptake by macrophages by mechanisms other than phagocytosis.

## Discussion

We previously demonstrated that angiopoietin-like 4 (ANGPTL4) is expressed by astrocytes in white matter and in gray matter of patients suffering from capillary cerebral amyloid angiopathy (8). Here, we show that the astrocytic expression of ANGPTL4 is markedly reduced in active demyelinating MS lesions compared to surrounding normal appearing white matter (NAWM). ANGPTL4 is a potent inhibitor of lipoprotein lipase (LPL), an enzyme involved in lipid uptake. We provide evidence that ANGPTL4 inhibits uptake of myelin-derived lipids by LPL-immunoreactive phagocytes. This finding suggests that loss of astrocytic ANGPTL4 expression in active demyelinating MS lesions might be a protective mechanism enabling phagocytes to effectively remove myelin debris paving the way for repair.

Phagocytes, i.e., microglia and macrophages, play a critical role in pathogenesis of MS. In MS, phagocytes promote the clearance of cellular debris after myelin damage, which is a prerequisite for remyelination (14, 15). Our work suggests a novel concept how astrocytes, via decreased production of ANGPTL4, regulate clearance of myelin debris. Reduced astrocytic expression of ANGPTL4 in active lesions might enhance LPL activity on phagocytes, thereby enabling the processing and subsequent clearance of myelin debris. However, full global knockout of ANGPTL4 in mice did not show any significant differences in LPL activity in the brain (16). This might be explained by the lack of LPL protein expression in control white matter brain tissue (17). LPL mRNA expression was almost exclusively detected in microglia/macrophage and specifically in alternatively activated microglia/macrophages (18, 19). Although microglia and macrophage activation states are complex, in particular in MS where they obtain an intermediate activation status (11), it appears that lipid-laden microglia/macrophages are immunosuppressive (20) which corresponds to an alternative activation status. We therefore opted to use alternatively activated macrophages in this study.

We next investigated which factors might be responsible for the decreased expression of ANGPTL4 on astrocytes in active MS lesions. We first cultured astrocytes in the presence of myelin debris, which has been shown to induce a reactive astrocyte profile (21) However, the expression of ANGPTL4 was not affected by myelin uptake. Astrocytes cultured in the presence of alternatively stimulated macrophages showed a significant reduction in the expression in astrocytic ANGPTL4. It thus appears that crosstalk between alternatively activated macrophages and astrocytes is involved in the downregulation of astrocytic ANGPTL4. Future studies are needed to gain more insight in the mechanisms how macrophages regulate astrocytic ANGPTL4 production.

Recent reports have highlighted the pivotal role of microglial LPL in remyelination. LPL hydrolyses triglycerides into glycerol and free fatty acids (FFA). The resulting FFAs can in turn be taken up via scavenger receptors, such as CD36 and CD68 by microglia (22). These scavenger receptors have been suggested to involved in myelin phagocytosis and have been shown to be upregulated in and around demyelinating areas in MS tissue (13).

Upon exposure to myelin fragments, the lipid storage in macrophages was dramatically increased as determined by Oil-Red-O staining. Inhibition of LPL in macrophages by addition of recombinant ANGPTL4 or the LPL inhibitor Orlistat significantly reduced the amount of Oil-Red-O positive lipid droplets. Interestingly, we did not observe differences in their capacity to phagocytose myelin. These findings suggest that ANGPTL4 inhibits the breakdown and subsequent uptake of myelin derived lipids by macrophages, but not myelin uptake via phagocytosis. In line with these observations, ANGPTL4 mediated inhibition of LPL has been shown to limit the formation of foam cells in the context of atherosclerosis (23, 24).

In conclusion, we provide evidence that ANGPTL4 expression is strongly reduced in reactive astrocytes in active MS lesions, which might be a protective mechanism enabling phagocytes to effectively remove myelin debris setting the stage for repair.

## Ethics Statement

VUmc MS Centrum Amsterdam and the Netherlands Brain Bank received permission to perform autopsies for the use of tissue and for access to medical records for research purposes from the Ethical Committee of the VU University Medical Center, Amsterdam, The Netherlands. All patients and controls, or their next of kin, had given informed consent for autopsy and use of brain tissue for research purposes. Buffy coats were obtained from volunteer donors at Sanquin after written informed consent was obtained.

## Author Contributions

AK with the help of JvH and HdV conceived the study. AK, JvH, MR, and HdV designed the experiments. AK with the help of AC performed most of the experiments. SvdP performed FACS experiments. AK with the help of JvH, MR, and HdV wrote the manuscript, which was reviewed by all authors.

### Conflict of Interest Statement

The authors declare that the research was conducted in the absence of any commercial or financial relationships that could be construed as a potential conflict of interest.

## Supplementary Material

Supplementary Figure 1 Gating strategy. Non-myelin treated monocyte derived macrophages were gated on forward scatter/side scatter (FCS/SSC) dot plot. These events were next visualized using an FSC/myelin-633 dot plot and a gate was placed for atto6330-labeled myelin negative cells. Then atto633-labeled myelin treated monocyte derived macrophages were analyzed to confirm that treated macrophages lay inside the gate.
