# Supplementary figures and images for: Reduced Angiopoietin-Like 4 Expression in Multiple Sclerosis Lesions Facilitates Lipid Uptake by Phagocytes via Modulation of Lipoprotein-Lipase Activity
Source: Front Immunol. 2019 May 3;10:950. doi: 10.3389/fimmu.2019.00950 (PMC6509157; doi:10.3389/fimmu.2019.00950)

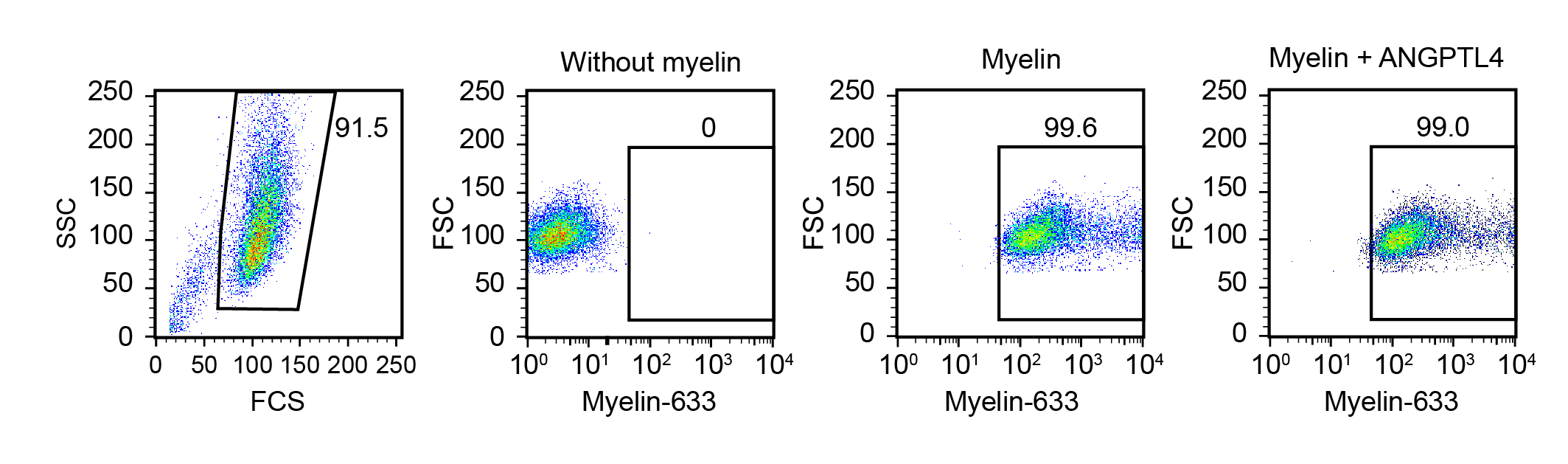

Supplement: Supplementary Figure 1 — Gating strategy. Non-myelin treated monocyte derived macrophages were gated on forward scatter/side scatter (FCS/SSC) dot plot. These events were next visualized using an FSC/myelin-633 dot plot and a gate was placed for atto6330-labeled myelin negative cells. Then atto633-labeled myelin treated monocyte derived macrophages were analyzed to confirm that treated macrophages lay inside the gate. [file Image_1.TIF]
